# Immunogenetic Architecture of Chronic Lymphocytic Leukemia at Early Stage: Insights from the O-CLL1 Cohort

**DOI:** 10.3390/antib15020025

**Published:** 2026-03-18

**Authors:** Davide Bagnara, Andrea Nicola Mazzarello, Monica Colombo, Ennio Nano, Niccolò Cardente, Fabiana Ferrero, Nadia Bertola, Vanessa Cossu, Fabio Ghiotto, Adalberto Ibatici, Emanuele Angelucci, Antonino Neri, Massimo Gentile, Fortunato Morabito, Manlio Ferrarini, Giovanna Cutrona, Franco Fais

**Affiliations:** 1 Department of Experimental Medicine, University of Genoa, 16132 Genoa, Italy; davide.bagnara@unige.it (D.B.); andreanicola.mazzarello@edu.unige.it (A.N.M.); fabiana.ferrero@edu.unige.it (F.F.); vanessa.cossu@edu.unige.it (V.C.); fabio.ghiotto@unige.it (F.G.); ferrarini.manlio@gmail.com (M.F.); 2 Molecular Pathology Unit, IRCCS Azienda Ospedaliera Metroplitana, 16132 Genoa, Italy; monica.colombo@hsanmartino.it (M.C.); ennio.nano@hsanmartino.it (E.N.); nadia.bertola@hsanmartino.it (N.B.); giovanna.cutrona@hsanmartino.it (G.C.); 3 Department of Immunology, University of Oslo, 0424 Oslo, Norway; nick.cardente99@gmail.com; 4 Hematology and Cellular Therapy Unit, Azienda Ospedaliera Metroplitana, 16132 Genova, Italy; adalberto.ibatici@hsanmartino.it (A.I.); emanuele.angelucci@hsanmartino.it (E.A.); 5 Scientific Directorate, Azienda USL-IRCCS di Reggio Emilia, 42122 Reggio Emilia, Italy; antonino.neri@ausl.re.it; 6 Hematology Unit, Department of Onco-Hematology, AO of Cosenza, 87100 Cosenza, Italy; m.gentile@aocs.it; 7 Department of Pharmacy, Health and Nutritional Science, University of Calabria, 87036 Rende, Italy; 8 Gruppo Amici Dell’Ematologia Foundation-GrADE, 42100 Reggio Emilia, Italy; f.morabito53@gmail.com

**Keywords:** chronic lymphocytic leukemia, IGHV repertoire, B-cell receptor stereotypy, early-stage CLL, Immunogenetics, antigen-driven selection

## Abstract

Background/Objectives: The immunoglobulin heavy-chain variable (IGHV) gene repertoire represents a characteristic feature of chronic lymphocytic leukemia (CLL), although its configuration is not well defined at the early disease stages. The IGHV repertoire of a cohort of early CLL patients was analyzed and compared to that of a “real-world” reference cohort. Methods: Patients from the O-CLL1 observational protocol, which enrolled only Binet stage A cases within twelve months from diagnosis, were studied. IGHV/IGHJ rearrangements were sequenced and annotated following ERIC recommendations, and stereotyped subsets were assigned using ARResT/AssignSubsets. The repertoire features were compared with the dataset of a real-world cohort of patients with heterogeneous staging (CTR cohort) and with published early-diagnosis series. Results: IGHV and IGHJ gene distributions and HCDR3-length profiles in O-CLL1 closely mirrored those of CTR, indicating that the BcR IG repertoire at diagnosis is already defined rather than being selected during disease progression. Mutated IGHV (M-CLL) predominated, with a frequency of stereotyped BcR IG comparable to that of other early-diagnosis cohorts. However, within this conserved framework, subset #4 was over-represented among M-CLL from O-CLL without an increased overall IGHV4-34 gene usage, suggestive of a selective expansion rather than a recombinational bias. Subset #4 cases retained canonical HCDR3 motifs and showed time-to-first-treatment like other M-CLL, likely reflecting the younger age structure of O-CLL1. Conclusions: Early-diagnosis CLL displays a biased IGHV repertoire with stereotyped configurations characteristic of CLL, including subsets that are rare in the normal B-cell repertoire. These findings support a central role for antigen-driven selection in shaping CLL evolution.

## 1. Introduction

Chronic lymphocytic leukemia (CLL) is a lymphoid malignancy characterized by the accumulation of monoclonal B lymphocytes with a characteristic immunophenotype—typically CD5^+^ and CD23^+^, CD22^−^, and low levels of surface immunoglobulins. Despite this relative immunophenotypic uniformity, CLL patients exhibit highly variable clinical courses, which reflect distinct biological and genetic backgrounds, including specific gene mutations and chromosomal abnormalities [1,2].

The mutational status of the immunoglobulin heavy-chain variable (IGHV) gene represents one of the strongest prognostic markers in CLL. Patients with unmutated IGHV genes generally experience a more aggressive disease and a shorter survival, whereas those with mutated IGHV genes have a more indolent course [3,4]. In addition, the CLL B-cell receptor (BcR IG) repertoire is notably biased, with certain IGHV, IGHD, and IGHJ segments being preferentially used within the mutated or unmutated subgroups [5].

A striking feature of the CLL immunoglobulin repertoire is the existence of stereotyped BcR IG [6]. Distinct CLL clones may express quasi-identical receptors sharing highly homologous heavy-chain complementarity-determining region 3 (HCDR3) sequences and, often, nearly identical IGHV amino acid sequences. These similarities frequently extend to the light-chain rearrangements as well [7,8,9]. The discovery of stereotyped BcR IG provided strong evidence that antigen-driven selection contributes to leukemic clonal expansion.

Stamatopoulos et al. demonstrated that approximately 20% of CLL cases can be grouped into stereotyped subsets defined by highly homologous HCDR3 motifs and the use of IGHV genes belonging to the same phylogenetic clan [10]. Among these, those represented by at least three independent cases were considered “confirmed,” while the most recurrent and biologically distinct became known as major stereotyped subsets. In some instances, these are characterized by reproducible HCDR3 motifs and distinctive IGHV/IGLV pairings, such as subset #1 (IGHV1-2/1-3/5a + IGKV1-39), subset #2 (IGHV3-21 + IGLV3-21), or subset #4 (IGHV4-34 + IGKV2-30). Subsequent work by the ERIC consortium further refined the concept [11,12]. In addition, more recently, Agathangelidis et al. reported that, in a cohort of nearly 30,000 cases, >40% of CLL patients carry a stereotyped BcR IG, with 29 major subsets accounting for ~13.5% of all cases [12]. Collectively, these findings show that stereotyped BcR IG delineate biologically distinct disease variants within CLL and underscore the central role of antigenic selection in CLL pathogenesis. This concept is reinforced by the observation of a close correlation between the stereotyped BcR IG subset and the clinical course as reviewed in [13].

Mapping the immunogenetic repertoire at the earliest clinically recognized stages of CLL is relevant for both biological and clinical reasons. From a biological perspective, defining which IGHV genes and stereotyped BcR IG configurations are already present at diagnosis helps position early CLL along the MBL–CLL continuum and clarifies whether key repertoire features arise before or after overt leukemic transformation. Clinically, because IGHV status and BcR IG stereotypy are tightly linked to prognosis and, in some subsets, to patterns of disease evolution and treatment response, a detailed characterization of the early-stage repertoire may refine risk stratification beyond the binary mutated/unmutated classifier in patients who are otherwise managed with a watch-and-wait approach. Importantly, in the current therapeutic landscape there is no evidence-based indication to initiate treatment in asymptomatic early-stage CLL before disease progression [14], even in the presence of high-risk biomarkers; therefore, the immediate clinical utility of early immunogenetic profiling is not to trigger therapy, but rather to improve prognostic counseling, guide the intensity and focus of monitoring, and provide a rational framework for designing and interpreting longitudinal studies and future early-intervention trials. In addition, delineating which stereotyped configurations are already “fixed” at presentation versus those that emerge later may have implications for the timing and targeting of BCR-directed interventions, should early-treatment strategies become justified by forthcoming evidence.

Most studies on IGHV repertoire and stereotyped BcR IG usage have examined patients across all disease stages. Consequently, a detailed analysis of the immunogenetic repertoire at the early stages of CLL is lacking. In the present work, we address this issue by analyzing the IGHV repertoire of CLL patients enrolled in the O-CLL1 observational study within twelve months from diagnosis [15]. This early-diagnosis cohort thus provides a unique opportunity to investigate the immunogenetic landscape and to examine how stereotyped configurations, including those rare in healthy individuals, are already established at disease presentation.

## 2. Materials and Methods

### 2.1. Study Design and Patient Cohort

The O-CLL1 (Observational CLL 1) study is a prospective, multicenter observational trial initiated by the Gruppo Italiano Studio Linfomi (GISL). Detailed information has been reported elsewhere [15]. Briefly, the primary aim of the study (clinicaltrials.gov identifier: NCT00917540) was to prospectively investigate the value of molecular, cytogenetic, and clinical prognostic factors in patients with early-stage CLL. The O-CLL1 trial enrolled untreated Binet stage A CLL patients from more than 40 Italian institutions within twelve months of diagnosis. A total of 475 patients were included, with a median time from diagnosis to enrollment of 2.3 months. The enrollment period extended from January 2007 to January 2012. The median age at enrollment was 61.2 years, and 41% of patients were female. Patients older than 70 years were not eligible for O-CLL1 in order to maximize the likelihood of an adequate follow-up duration for the vast majority of enrolled individuals, thereby ensuring robust assessment of time-to-first-treatment and long-term outcome. Follow-up data were available for most patients, with a median follow-up duration of approximately 86 months. Time to first treatment (TTFT) was recorded for 470 patients; at the time of analysis, 179 (38%) had progressed and required therapy.

### 2.2. Biological Markers

Biological markers assessed at baseline included CD38 and ZAP-70 expression, NOTCH1 and SF3B1 mutations, and serum β2-microglobulin levels. TP53 mutations were analyzed by Sanger or next-generation sequencing (NGS) in all available cases (*n* = 470), and del(17p) was assessed by fluorescence in situ hybridization (FISH) in 469 patients.

#### 2.2.1. CTR Cohort

The control (CTR) cohort was defined by CLL cases collected and sequenced by Sanger for IGHV rearrangement at the IRCCS Azienda Ospedaliera Metropolitana (Genoa, Italy) and at the Feinstein Institutes for Medical Research, Manhasset (NY, USA). These cohorts were collected over the past 30 years and comprise 1933 IGHV rearrangements derived from patients across all disease stages (Table S1).

#### 2.2.2. CLL Stereotype Assignment

Rearranged IGHV-D-JH segments were amplified and sequenced from cDNA generated from total RNA isolated from peripheral blood mononuclear cells (PBMC); 1 μg RNA was reverse-transcribed with M-MLV reverse transcriptase (Thermo Fisher Scientific, Waltham, MA, USA) in the presence of RNase inhibitor and oligo(dT) primers (both from Thermo Fisher Scientific). IGHV family usage was assessed by PCR with IGHV family–specific leader primers paired with constant-region primers (Thermo Fisher Scientific). PCR products were purified and sequenced on an ABI 3730 DNA Analyzer (Applied Biosystems, Foster City, CA, USA); when mutations were detected, sequencing was confirmed on an independent PCR product [5]. CLL stereotyped sequences were identified by submitting IGHV-D-J sequences to ARResT/AssignSubsets (https://bat.infspire.org/arrest/assignsubsets/, accessed on 30 April 2025) [16].

#### 2.2.3. Statistical Analysis and Figures

Statistical analyses were performed in R (V 4.5.0, R Foundation for Statistical Computing, Vienna, Austria), using the tidyverse ecosystem (e.g., dplyr, tidyr) for data handling and ggplot2 for data visualization. Differences in the proportion of mutated (M-CLL) versus unmutated (U-CLL) IGHV cases between cohorts were assessed by Pearson’s chi-square test of independence. For IGHV gene usage and the frequency of individual stereotyped subsets, comparisons were performed within M-CLL and U-CLL strata by testing whether the observed count in the O-CLL1 cohort deviated from the corresponding CTR reference proportion using two-sided exact binomial tests. Time-to-first-treatment (TTFT) and overall survival (OS) were estimated by Kaplan–Meier methods and compared between groups by log-rank tests. Multiple testing arising from per-gene/per-subset analyses was controlled using the Benjamini–Hochberg false discovery rate procedure. Age distributions were compared using pairwise Wilcoxon rank-sum test. All tests were two-sided and *p* < 0.05 was considered statistically significant.

## 3. Results

### 3.1. IGHV Repertoire in the O-CLL1 and CTR Cohorts

We compared the IGHV gene repertoire of the O-CLL1 cohort with a collection of “real-world” IGHV rearrangements obtained from two single-center cohorts (see Section 2), collectively defined as the CTR cohort.

The representativeness of the CTR cohort was assessed by comparing key repertoire parameters with the values reported by Stamatopoulos et al. [10], including IGHV gene usage, the proportion of mutated IGHV genes (>2% nucleotide mutations), and the frequency of stereotyped BcR IG. All three parameters showed close agreement between the two datasets: the proportion of mutated IGHV genes was 54% in our cohort versus 55% in the reference, and the frequency of stereotyped BcR IG was 11.7% versus 12.3%, respectively. Therefore, the CTR cohort was used as a validated reference for immunogenetic comparisons.

**Figure 1:**
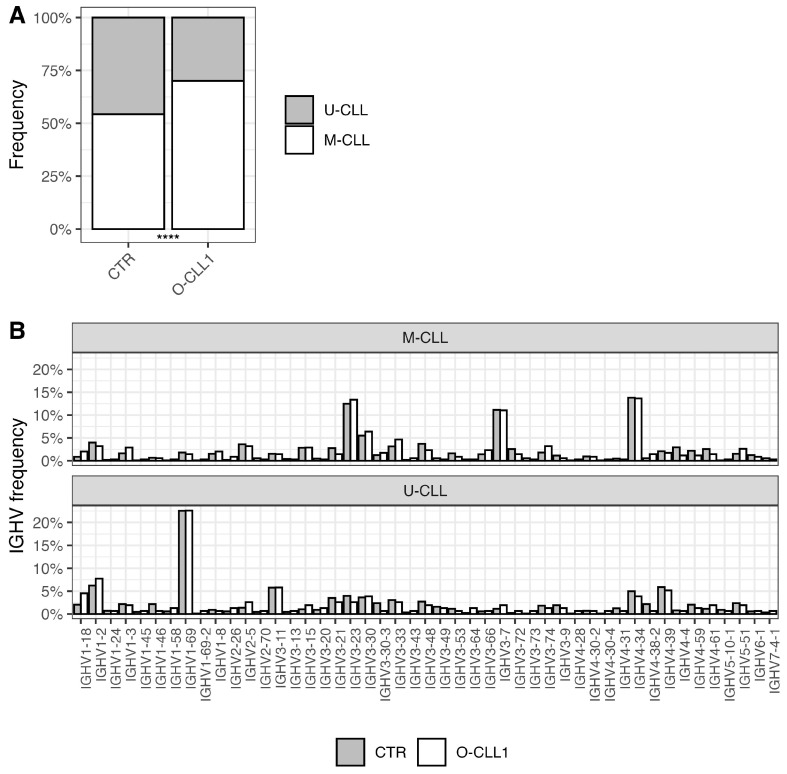
(**A**) Distribution of mutated (M) and unmutated (U) immunoglobulin heavy-chain variable region (IGHV) status in the CTR cohort and O-CLL1. A chi-square test showed a statistically significant difference between cohorts (**** *p* < 0.0001). (**B**) IGHV gene usage in M-CLL and U-CLL within the CTR cohort and O-CLL1. Binomial tests performed for each IGHV gene–mutation status combination did not reveal any statistically significant differences between cohorts.

In the O-CLL1 cohort, mutated IGHV cases represented approximately 71% of successfully sequenced rearrangements, compared to 54% in the CTR cohort (Figure 1A, *p* < 0.0001). Given this imbalance, subsequent analyses were conducted separately for mutated (M) and unmutated (U) cases, as the IGHV repertoire and the representation of stereotyped BcR IGs differ between M-CLL and U-CLL [5,10,11].

As shown in Figure 1B, the overall distribution of IGHV genes was highly similar between O-CLL1 and CTR. This concordance was especially evident for major IGHV genes frequently used in CLL, namely IGHV1-69, IGHV4-34, IGHV3-21, and IGHV3-7. IGHJ gene usage (Supplementary Figure S1A) and the HCDR3-length distribution (Supplementary Figure S1B) were also largely superimposable between cohorts.

### 3.2. Representation of Stereotyped BcR IG Subsets

**Figure 2:**
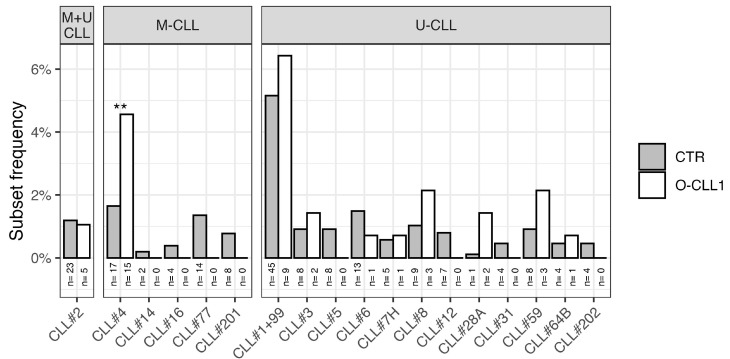
Frequency of individual CLL stereotyped subsets in mutated (M-CLL), unmutated (U-CLL), and, where applicable, combined cases (subset #2) in the CTR and O-CLL1 cohorts. Binomial tests were performed; only statistically significant results are indicated (** *p* ≤ 0.01).

Major stereotyped subsets were identified using ARResT/AssignSubsets [16]. Because stereotyped subsets are strongly associated with IGHV mutational status and the datasets differ in the proportion of mutated sequences, analyses were conducted separately for mutated and unmutated CLL. The only exception was subset #2, which includes cases from both categories (Figure 2) and was therefore analyzed as a fraction of total cases, considering both mutated and unmutated rearrangements.

The distribution of major stereotyped subsets in unmutated CLL cases did not differ substantially between the two cohorts. However, among mutated cases, subset #4 was significantly over-represented in the O-CLL1 cohort (*p* < 0.01), whereas other mutated subsets (excluding subset #2) were not observed in O-CLL1, although the difference compared with CTR did not reach statistical significance. The frequency of subset #2 among all cases was consistent with the CTR cohort (Figure 2). Importantly, despite the enrichment of subset #4, the underlying IGHV4-34 gene usage in mutated O-CLL1 cases was not increased.

**Figure 3:**
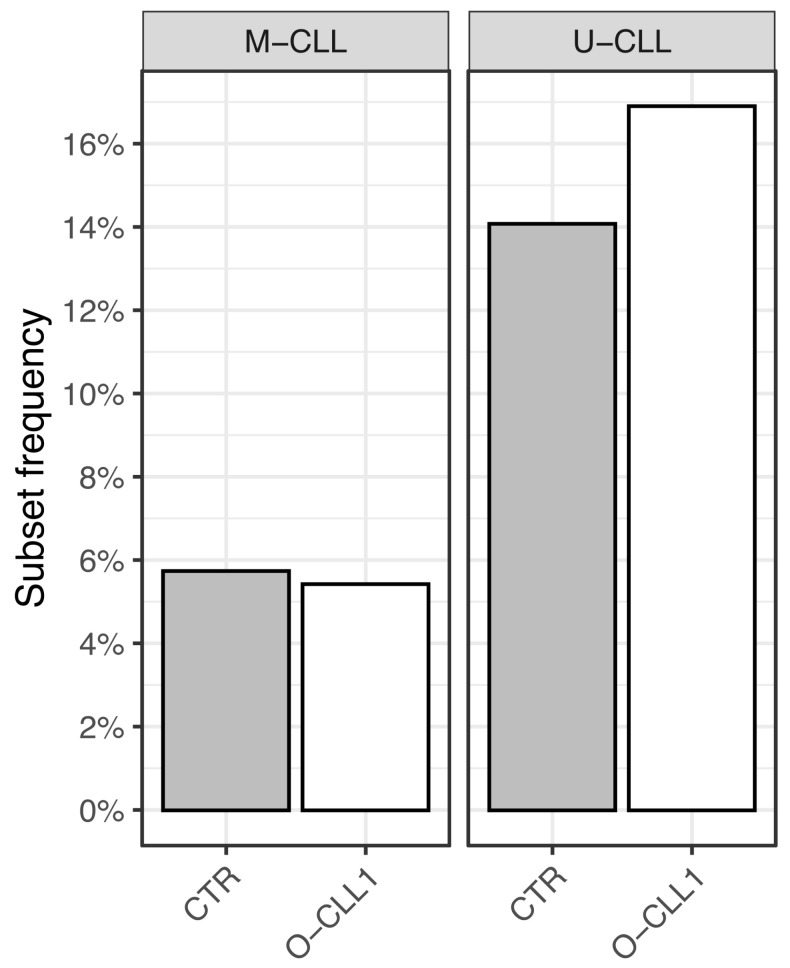
Frequency of major CLL stereotyped subsets (indicated as Subset in the figure) in mutated (M-CLL) and unmutated (U-CLL) cases from the CTR and O-CLL1 cohorts. No statistically significant differences were detected by binomial testing.

Among unmutated cases, the overall frequency of stereotyped subsets was similar between cohorts, with minor differences limited to rarely encountered subsets (Figure 3).

### 3.3. Features of Subset #4 Cases of O-CLL1

Subset #4 is classically associated with a highly mutated IGHV4-34 configuration and an indolent clinical course [10,17]. The HCDR3 amino acid composition of subset #4 in O-CLL1 was substantially identical to that of subset #4 in the CTR cohort (Supplementary Figure S2), confirming full immunogenetic consistency. Cytogenetically, 5 of 15 subset #4 cases carried a 13q deletion as the sole abnormality, in line with the favorable-risk features typically associated with this subset [10,17].

**Figure 4:**
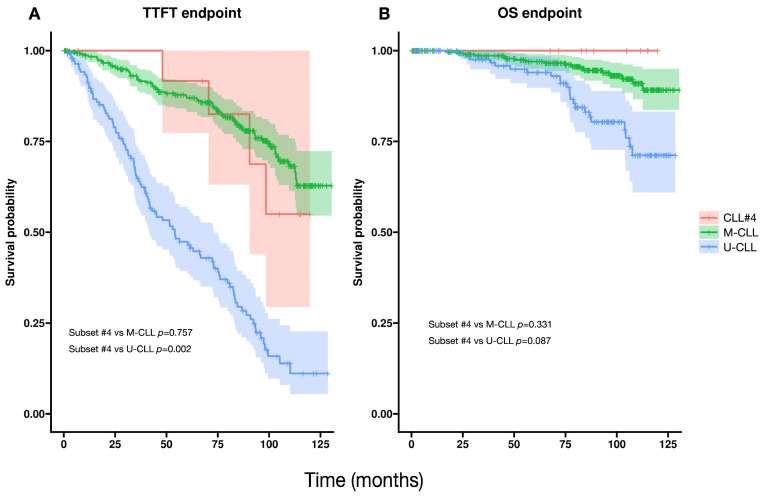
Clinical outcomes in the O-CLL1 cohort according to IGHV mutational status and the presence of stereotyped subset #4. (**A**) Kaplan–Meier curves for time to first treatment (TTFT) in subset #4 CLL cases (*n* = 15), mutated CLL (M-CLL; *n* = 124), and unmutated CLL (U-CLL; *n* = 331). Group differences were assessed using two-sided log-rank tests; subset #4 differed significantly only from U-CLL (log-rank *p* = 0.002). (**B**) Kaplan–Meier curves for overall survival (OS) in subset #4 CLL cases (*n* = 15), M-CLL (*n* = 330), and U-CLL (*n* = 122). Overall survival for subset #4 did not differ significantly from that of M-CLL (log-rank *p* = 0.087).

In the O-CLL1 cohort, subset #4 cases exhibited a time-to-first-treatment (TTFT) comparable to that of the overall mutated group (Figure 4). Although the overall survival (OS) curve for subset #4 did not reach statistical significance when compared with other M-CLL cases, the trend indicates a potential divergence over longer follow-up.

**Figure 5:**
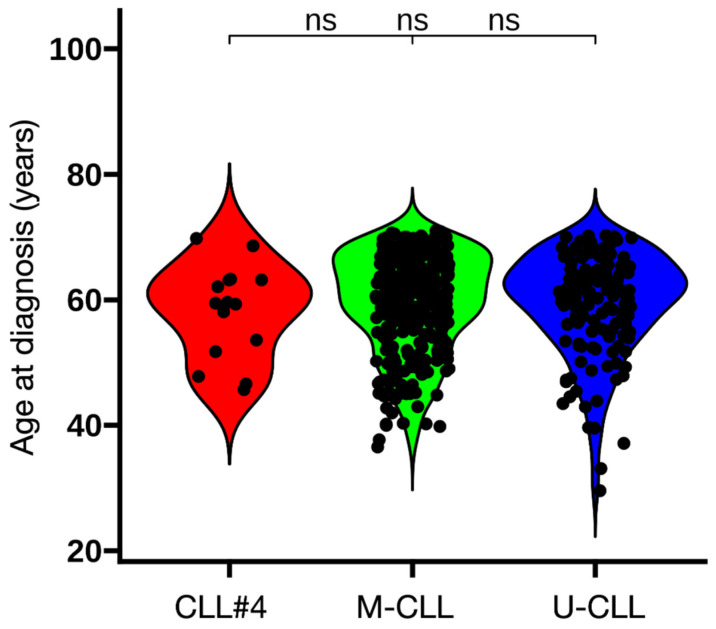
Violin plots showing age at diagnosis for patients belonging to subset #4 compared with mutated (M) and unmutated (U) CLL cases of the O-CLL1 cohort. The violin shape depicts the distribution/density of ages in each group, with black dots indicating individual patients. The colors in the violin plots are used solely to improve the visual distinction between the different groups. Subset #4 CLL cases (*n* = 15), M-CLL (*n* = 124), and U-CLL (*n* = 331) are shown. Age distributions were compared using the pairwise Wilcoxon rank-sum test; “ns” indicates *p* > 0.05.

We also evaluated the age at diagnosis of subset #4 patients, as this subset has been reported to present at a younger age [10,17]. In the O-CLL1 cohort, however, no differences were observed when compared with M-CLL and U-CLL (Figure 5).

## 4. Discussion

The analysis of the O-CLL1 cohort provides an immunogenetic snapshot of CLL at its earliest clinically recognized stage. The IGHV gene repertoire of these newly diagnosed patients closely mirrored that of the CTR cohort, indicating that the core architecture of the CLL BcR IG repertoire is already established at presentation and is not substantially distorted by early sampling or diagnostic timing.

In the pre-leukemic setting, earlier immunogenetic studies comparing monoclonal B-cell lymphocytosis (MBL) with overt CLL reported substantial differences in IGHV gene usage, lower frequencies and diversity of stereotyped subsets, and a mutated-to-unmutated ratio skewed toward mutated rearrangements, with high-count MBL appearing more similar to CLL than low-count MBL [18,19,20]. More recently, high-throughput, next-generation sequencing analyses of low-count and high-count CLL-type MBL have revealed a more composite picture [21], in which each case harbors a complex clonal architecture that already includes BcR IG configurations typical of CLL, biased usage of “canonical” CLL IGHV genes and a sizable fraction of stereotyped receptors, embedded within a broader, less consolidated repertoire. Across this spectrum, from low-count to high-count CLL-type MBL and finally to overt CLL, the immunogenetic signature appears to “mature” through progressive consolidation of a single dominant clone and increasing intraclonal diversification, rather than through the abrupt acquisition of an entirely new BcR IG repertoire. Within this maturation framework, the O-CLL1 cohort most likely captures a disease stage in which a CLL-like dominant clone has already emerged and consolidated within an immunogenetically biased background. This is consistent with the close concordance we observed between O-CLL1 and the CTR cohort in terms of IGHV/IGHJ usage and HCDR3 length distribution, indicating that most repertoire-level features already resemble those of established CLL. At the same time, some differences remain, including the skewed mutated-to-unmutated IGHV ratio in O-CLL1 and the relative enrichment of stereotyped subset #4 within the mutated subgroup. The latter feature may reflect both biological aspects of early-diagnosis CLL and characteristics of the study design, such as the upper age limit of 70 years, which could favor the inclusion of patients with mutated and clinically indolent immunogenetic categories. Therefore, caution is warranted when extrapolating the specific subset distribution observed in O-CLL1 to unselected CLL populations that include older individuals.

One likely explanation for the predominance of mutated IGHV in early-diagnosis cohorts such as O-CLL1 is the different time spent in the asymptomatic, early-stage phase of mutated versus unmutated disease in the asymptomatic, early-stage phase. Mutated CLL typically progresses more slowly and can remain in a Binet A/watch-and-wait state for many years, thereby being preferentially captured by studies that enroll patients shortly after diagnosis. In contrast, unmutated cases often traverse this early window more rapidly and are consequently under-represented among early-stage patients but become relatively more frequent in mixed-stage or treatment-oriented series, where the cumulative burden of biologically aggressive clones is enriched.

Thus, the O-CLL1 IGHV repertoire more closely resembles fully established CLL, supporting the notion that most immunogenetic features of CLL are already established at clinical presentation.

Consistent with established CLL series, IGHV usage in O-CLL1 was dominated by IGHV1-69, IGHV4-34, IGHV3-21, and IGHV3-7, in line with the biased repertoire originally described by Fais and colleagues and later confirmed in large immunogenetic datasets [5,10,11]. The close overlap with the CTR cohort across IGHV/IGHJ usage and HCDR3 length distribution indicates that recombinational bias and antigen-driven selection are already active at diagnosis and remain globally stable thereafter.

Our findings are also consistent with recent early-diagnosis studies in de novo CLL cohorts from Southern Europe and Asia reported a predominance of mutated IGHV genes and a modest but reproducible frequency of stereotyped BcR IG, with geographic variability in the representation of subsets such as #4 and #8 [22,23]. Thus, a repertoire enriched for mutated IGHV and containing a restricted but stable set of CLL-biased stereotyped configurations appears to be a conserved feature of CLL at diagnosis.

Within this framework, the most distinctive finding in O-CLL1 is the disproportionate representation of subset #4 within the mutated subgroup. Subset #4 is an archetypal highly mutated subset associated with IGHV4-34 and, in most published cohorts, with younger age at diagnosis and an indolent clinical course [10,17]. However, this age association was not observed in O-CLL1, as patients belonging to subset #4 did not differ in age from the mutated or unmutated groups (Figure 5). Importantly, IGHV4-34 usage itself was not increased in O-CLL1 compared with the CTR cohort, indicating that this skewing does not reflect a global expansion of IGHV4-34–expressing clones but rather a subset-specific enrichment.

Clinically, and in contrast with some published series [10,17], subset #4 cases in O-CLL1 displayed a TTFT comparable to that of the overall mutated group. OS also did not differ significantly, although a trend toward improved long-term outcome was observed (Figure 4). Their HCDR3 amino-acid composition and IGHV4-34 pairing were fully conserved (Supplementary Figure S2), confirming the immunogenetic identity of subset #4 in this cohort [10,11]. In addition, the cytogenetic profile of subset #4 cases was characterized by del(13q) as the sole abnormality in one-third of patients, further supporting a biologically indolent phenotype. The absence of age differences between subset #4 and the remaining mutated and unmutated cases may contribute to their clinical behavior being more similar to the mutated group in this specific cohort.

Our findings also align with high-throughput studies of the normal B-cell repertoire showing that several CLL-biased stereotyped rearrangements, especially subsets #4 and #8, are extremely rare or undetectable in healthy donors [24,25]. Their detectable representation in newly diagnosed CLL, including in O-CLL1, therefore argues against a simple reflection of their low baseline prevalence in the normal repertoire and supports the contribution of early selective pressures, such as autonomous [26,27], or specific microenvironmental interactions, to the expansion of these otherwise infrequent clonotypes [28,29,30,31,32,33].

From a broader immunogenetic perspective, stereotyped BcR IG are not confined to a single geographic or ethnic context. Large multicentre ERIC series and population-based cohorts from Europe and Asia consistently show that a substantial fraction of CLL cases carry stereotyped BcR IG and that the same major subsets (e.g., #1, #2, #4, #8) recur across different populations, albeit with quantitative differences in their relative frequencies [11,12,22,23,34]. These data argue against stereotyped BcR IG being race-restricted phenomena and instead support the notion of shared, globally relevant antigenic drivers (microbial, self, or quasi-self) acting on partially distinct germline IG gene pools and environmental backgrounds. In this light, the subset distribution observed in O-CLL1 likely reflects general principles of CLL ontogeny, even though the exact prevalence of individual subsets may vary according to geographic and demographic context.

These observations naturally raise the question of whether early-diagnosis cohorts could also be exploited to identify individuals at increased risk of developing CLL on the basis of adverse-risk stereotyped BcR IG configurations, such as subsets #2 and #8. Longitudinal repertoire studies in population-based cohorts have shown that dominant clonotypes belonging to high-risk stereotyped subsets, as well as unmutated CLL-like clonotypes, can be detected many years, up to 16 years, before overt CLL diagnosis [35], indicating that such immunogenetic profiles may mark a prolonged preclinical phase of disease evolution rather than only late events at transformation. However, it should be emphasized that, even when a skewed CLL-like repertoire or high-risk stereotyped clonotypes are detected, there is currently no indication to initiate treatment in asymptomatic early-stage CLL, as early therapy has not been shown to improve overall survival (reviewed in [36]), and current guidelines do not support treatment or screening based solely on prediagnostic immunogenetic findings [14]. In this context, the main near-term implication of detecting adverse-risk stereotyped BcR IG at early stages is to refine biological risk stratification and to inform the design of longitudinal studies aimed at distinguishing “progressing” from “stable” MBL/early CLL, rather than to mandate changes in clinical management in routine practice.

## 5. Conclusions

In summary, our analysis of the O-CLL1 cohort shows that, by the time CLL is first clinically recognized, the core immunogenetic architecture of the disease is already in place. Newly diagnosed, early-stage patients display a biased IGHV/IGHJ usage, characteristic HCDR3 length distribution, and a stable set of stereotyped BcR IG subsets that closely resemble those observed in established CLL. This underscores the importance of early antigenic selection and clonal dynamics in shaping CLL evolution.

## Data Availability

The dataset can be made available upon request to the authors.

## Supplementary Materials

The following supporting information can be downloaded at: https://www.mdpi.com/article/10.3390/antib15020025/s1, Figure S1: HCDR3 junction features of O-CLL1 cohort; Figure S2: Logos of HCDR3 aa composition in CLL#4 of CTR control and O-CLL1; Table S1: Main immunogenetic features of CLL cohorts.

## Abbreviations

The following abbreviations are used in this manuscript:

AIRC	Associazione Italiana per la Ricerca sul Cancro
BcR IG	B-cell receptor immunoglobulin
BCR	B-cell receptor
CLL	chronic lymphocytic leukemia
CTR	control/reference CLL cohort
ERIC	European Research Initiative on CLL
FISH	fluorescence in situ hybridization
GISL	Gruppo Italiano Studio Linfomi
HCDR3	heavy-chain complementarity-determining region 3
IGH	immunoglobulin heavy chain
IGHD	immunoglobulin heavy-chain diversity region
IGHJ	immunoglobulin heavy-chain joining region
IGHV	immunoglobulin heavy-chain variable region
IGKV	immunoglobulin kappa light-chain variable region
IGLV	immunoglobulin lambda light-chain variable region
MBL	monoclonal B-cell lymphocytosis
M-CLL	mutated IGHV chronic lymphocytic leukemia
NGS	next-generation sequencing
OS	overall survival
PBMC	peripheral blood mononuclear cells
PCR	polymerase chain reaction
SF3B1	splicing factor 3B subunit 1
TP53	tumor protein p53
TTFT	time to first treatment
U-CLL	unmutated IGHV chronic lymphocytic leukemia
VDJ	variable (V), diversity (D), and joining (J) gene segments
ZAP-70	zeta-chain-associated protein of 70 kDa
