# Supplementary figures and images for: Immunogenetic Architecture of Chronic Lymphocytic Leukemia at Early Stage: Insights from the O-CLL1 Cohort
Source: Antibodies (Basel). 2026 Mar 18;15(2):25. doi: 10.3390/antib15020025 (PMC13010646; doi:10.3390/antib15020025)

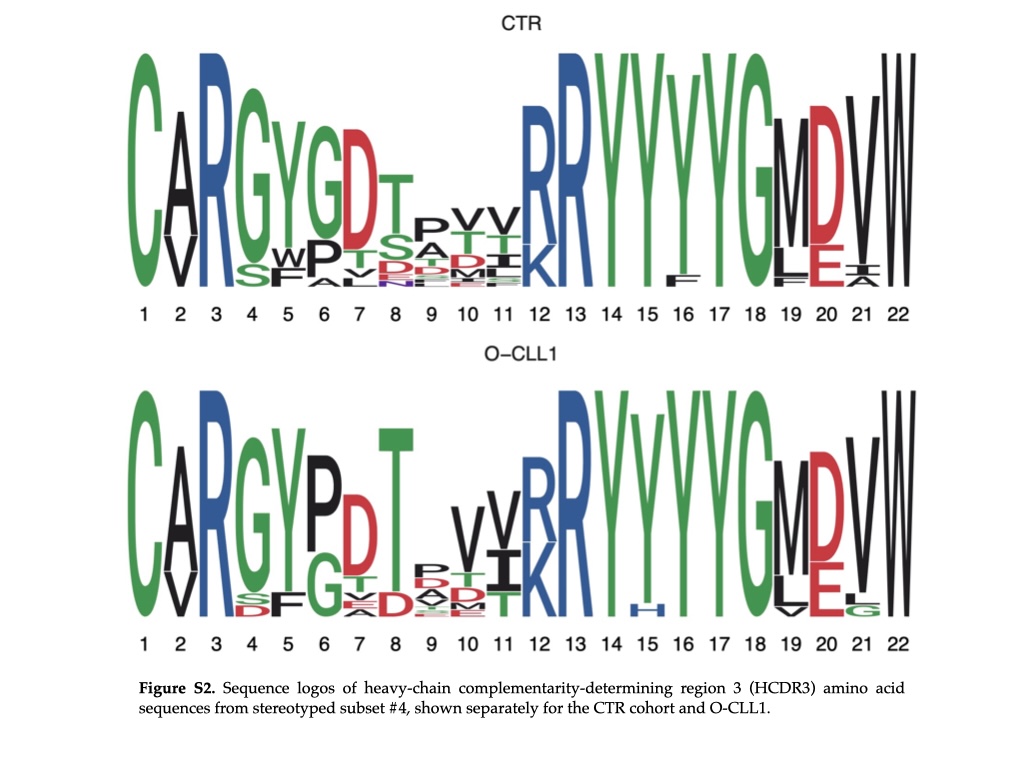

Supplement: Supplementary file 1 [file antibodies-15-00025-s001.zip › Figure S2.jpeg]

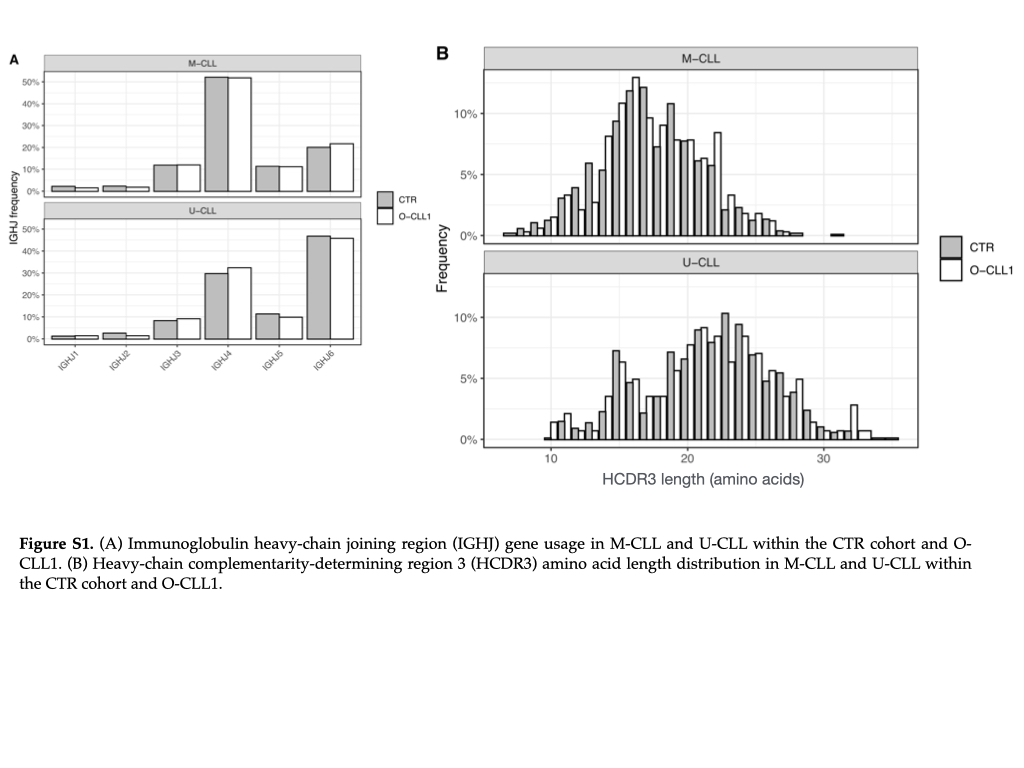

Supplement: Supplementary file 1 [file antibodies-15-00025-s001.zip › Figure S1.jpeg]
